# Primary extraskeletal osteosarcoma of the mesentery: A case report

**DOI:** 10.1016/j.ijscr.2019.05.058

**Published:** 2019-06-11

**Authors:** Shingo Ito, Yuichi Terado, Reiko Shimojima, Yoshiaki Hara, Kazuhiro Narita, Yuji Tachimori, Manabu Goto

**Affiliations:** a Department of Gastroenterological Surgery, Kawasaki Saiwai Hospital, Kanagawa, Japan; b Department of Pathology, Kawasaki Saiwai Hospital, Kanagawa, Japan

**Keywords:** Extraskeletal osteosarcoma, Mesentery, Laparoscopic surgery

## Abstract

**Introduction:**

Extraskeletal osteosarcoma is a rare malignant soft tissue tumor without attachment to the bone. To the best of our knowledge, we present here the first report of a primary extraskeletal osteosarcoma of the mesentery in Japan.

**Case presentation:**

A 46-year-old female underwent a health examination, with no complaint. Following an ultrasonography a solid mass was detected in the abdominal cavity. Computed tomography showed a 38 × 25 mm heterogeneously enhancing mass. The latter was characterized by the presence of mottled calcifications and a cystic portion. The tumor was resected with a single incisional laparoscopic curative resection. Histopathological examination revealed the presence of a primary extraskeletal osteosarcoma arising from the mesentery. Postoperative course was uneventful. The patient did not receive chemotherapy during follow-up. She was recurrence free 10 months post-surgery.

**Discussion:**

Patients with extraskeletal osteosarcoma generally have a poor prognosis. A tumor size <5 cm represents an important prognostic factor. Unexpectedly, our case was detected by ultrasonography at an early stage. This is the first report of a single incisional laparoscopic resection.

**Conclusion:**

A primary extraskeletal osteosarcoma of the mesentery is an extremely rare occurrence. Its diagnosis should be taken into consideration also when a soft tissue mass of the mesentery is found.

## Highlights

• Extraskeletal osteosarcoma is a rare malignant soft tissue tumor without attachment to the bone.
• This is the first report of a single incisional laparoscopic resection.
• Its diagnosis should be taken into consideration also when a soft tissue mass of the mesentery is found.

## 1 Introduction

Primary abdominal extraskeletal osteosarcoma is extremely rare. It represents a malignant mesenchymal tumor, made of neoplastic cells that produce bone osteoid without attachment to bone [1]. Lower extremities are the most frequent location, followed by the upper limbs and retroperitoneum [2]. In the present report, we describe a case of an extraskeletal osteosarcoma of the mesentery. The SCARE criteria have been followed to report the present case [3].

## 2 Case presentation

**Fig. 1:**
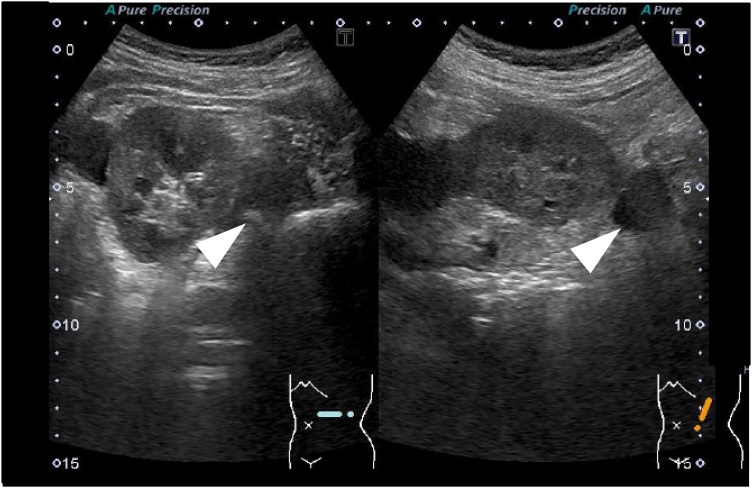
Abdominal ultrasonography shows the presence of a solid mass in close approximation to the kidney.

**Fig. 2:**
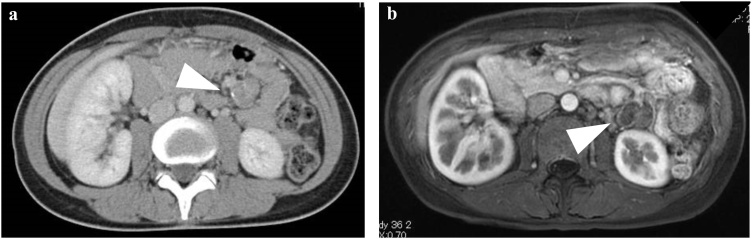
Enhanced CT scan shows a heterogeneously enhancing mass. The latter had mottled calcifications and a cystic portion. It measured 38 mm in length in abdominal cavity (a). T2-weighted MRI showed a low intensity mass separate from the bowel (b).

**Fig. 3:**
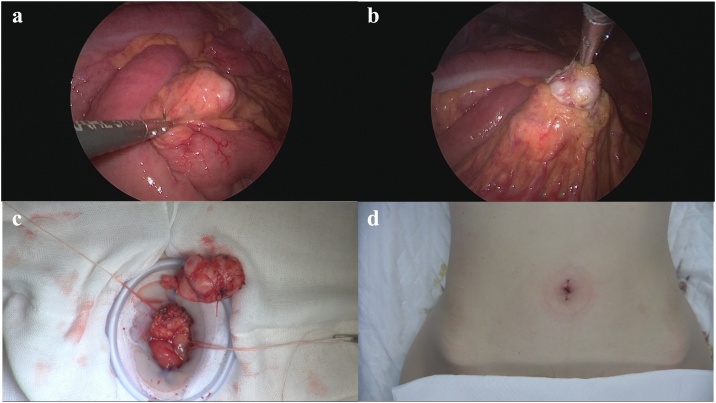
Intraoperative findings showed the presence of a 38 × 22 mm solid mass in the small bowel mesentery (a). *En bloc* mass excision with laparoscopic coagulation shears from mesentery (b). The tumor was completely resected (c). The umbilical incision (d).

**Fig. 4:**
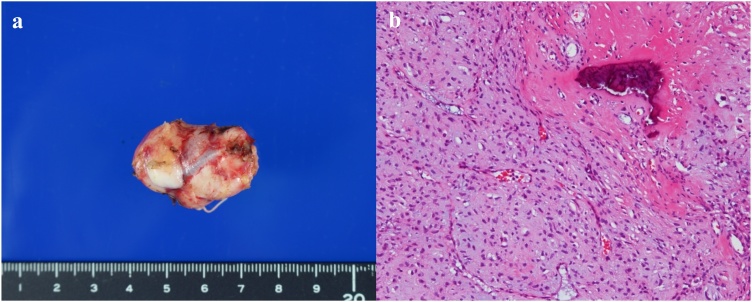
The resected specimen showed 38 × 25 × 13 mm elastic hard mass (a). Histopathologically, the tumor contained malignant tumor cells with osteoid formation (×100, H＆E) (b).

A 46-year-old female underwent a health examination with no complaint. No remarkable family history was reported. Her vital signs and blood tests were normal. Examination of the abdomen revealed no pain. However, following an abdominal ultrasonography, a solid mass was observed in close approximation to the kidney. The mass did not involve the abdominal cavity’s wall (Fig. 1). A further evaluation by computed tomography showed the presence of a 38 × 25 mm heterogeneously enhancing mass, with mottled calcifications and a cystic portion arising from small bowel mesentery (Fig. 2a). A low intensity mass of small bowel mesentery was observed by magnetic resonance imaging (T2 WI) (Fig. 2b). We diagnosed either sarcoma of the mesentery or gastrointestinal stromal tumor. The patient underwent a single incisional laparoscopic curative resection of the tumor. During the operation, the tumor was identified in the small bowel mesentery. It did not involve the stomach, intestine and marginal vessel (Fig. 3). The resected tumor measured showed 38 × 25 × 13 mm. Externally, the tumor had a smooth surface. Histopathological findings revealed nests of round to oval cells. The focal area showed the presence of more atypical cells with surround osteoid formation (Fig. 4). The final histologic diagnosis was of primary extraskeletal osteosarcoma arising from the mesentery. The patient underwent an uneventful postoperative course. She did not receive chemotherapy during her follow-up and had no recurrence 10 months post-surgery.

## 3 Discussion

Extraskeletal osteosarcoma is an extremely rare condition. It has been reported to account for about 1% of all soft tissue sarcomas and 4% of all osteosarcomas [4]. Extraskeletal osteosarcomas are most frequently found in the deep soft tissue of the thigh (47–68%) and less frequently in the upper extremity and retroperitoneum (12% each) [5]. Due to their localization, retroperitoneal and intraabdominal tumors have a delayed presentation. Additionally, they tend to be aggressive diseases with a poor prognosis [6]. In Japan, there have been reports of extraskeletal osteosarcoma arising from the retroperitoneum [7]. However, to the best of our knowledge, and based on a search of the English literature, ours is the first report of an extraskeletal osteosarcoma of the mesentery in Japan. The first report of an extraskeletal osteosarcoma dates back to 1941 [8]. It is generally seen in individuals >50 years old. The tumor has been reported to be associated with trauma, local radiotherapy, malignant fibrous tissue disease or myositis ossificans [9]. In the present case, a 46 years old woman no known risk factors. Extraskeletal osteosarcomas are more frequent in males than females. They present as fairly large masses, about 9 cm in size [10]. In an earlier study, Sio et al. [11] reported one of the earliest series of 37 patients with extraskeletal osteosarcomas. In a multivariate analysis, the authors observed that a primary size >10 cm was a significantly poor prognostic factor for overall survival. Additionally they found that a primary size >10 cm and older age were significantly influenced disease free survival with a worse outcome. Extraskeletal osteosarcoma of the mesentery is an extremely rare condition. The first case was reported in 1956 by Fine and Stout [12]. Including our case, there are only seven documented cases in the literature. Table 1 summarizes the latter reports. Specifically, it includes patient characteristics and demographics along with tumor information during initial diagnosis [2,[12], [13], [14], [15], [16]]. The median age of the seven patients (four males) was 46 years (range, 39–71 years). Three patients had tumors >10 cm clinically. Two of seven patients received postoperative chemotherapy. The present case was the first to be treated by laparoscopic resection. Three of the seven patients were alive.

**Table 1:** Literature review of extraskeletal osteosarcoma of the mesentery cases.

	Author (year)	Age	Sex	Size (cm)	Surgical procedure	Adjuvant therapy	Prognosis
1	Fine et al. (1956) [12]	39	M	–	open	unknown	Dead
2	Choudur et al. (2005) [2]	45	M	15	Open	Doxorubicin	Alive
						cisplatin	
3	Lee et al. (2007) [13]	67	M	15	Open	Ifosfamide	Dead
						adriamycin	
4	Heukamp et al. (2007) [14]	61	–	–	Open	–	–
5	Hussain et al. (2011) [15]	40	M	13	–	–	–
6	van den Broek et al. (2018) [16]	71	F	–	Open	none	Alive
							(peritoneal metastasis)
7	Our case (2018)	46	F	3.8	Laparoscopy	none	Alive

In earlier study by Allan et al. [4], the authors showed the criteria for the diagnosis for primary extraskeletal osteosarcoma. As follows: presence of a uniform morphological pattern of sarcomatous tissue excluding the possibility of malignant mesenchymoma, production of malignant osteoid or bone by the sarcomatous tissue, and ready exclusion of an osseous origin. On CT, the tumor is characterized by the presence of a calcified mass with areas of soft tissue attenuation, with no osseous involvement [10]. On MRI imaging, the tumor shows a nonspecific intermediate signal intensity on T1-weight imaging and high signal intensity on T2-weight imaging, and enhances with gadolinium [17]. A wide surgical resection represents the treatment of choice for patients with an extraskeletal osteosarcoma. However, identification of the optimal treatment of extraskeletal osteosarcomas remains to date a challenge. Depending on differences in development, location and range of the disease, a wide excision, radical resection or simple excision may be used as needed. In a previous study it has been suggested that wide resection is effective for extraskeletal osteosarcoma. The authors showed an improved prognosis compared with simple resection [18]. Furthermore, the recurrence ratio or metastasis may be reduced by neoadjuvant radiotherapy or chemotherapy.

## 4 Conclusion

We report the case of a primary extraskeletal osteosarcoma of the mesentery. This condition is extremely rare. A preoperative diagnosis is difficult. However, in case of the presence of a tumor with calcific parts, the differential diagnosis for extraskeletal osteosarcoma should be taken into consideration. Laparoscopic surgery may be an effective treatment for relatively small extraskeletal osteosarcomas.

## Conflicts of interest

The authors have no conflicts of interests.

## Sources of funding

None.

## Ethical approval

This study was approved by the institutional review board, and written informed consent was obtained from the patient.

## Consent

Written consent was obtained from the patient for publication of this report.

Any details identifying the individuals to the clinical history and images associated were eliminated as to remain anonymous.

A copy of the written consent is available for review by the Editor-in-Chief of this journal on request

## Author’s contribution

Yuji Tachimori and Manabu Goto made substantial contributions to the conception and design. Yuichi Terado was responsible for the pathological finding. Kazuhiro Narita and Reiko Shimojima, Yoshiaki Hara were involved in surgical treatment. Yuji Tachimori made a critical revision of the article for important intellectual content.

All authors have read and approved the final version of the manuscript.

## Registration of research studies

This study registered at Research registry.

The registry number is 4728 at http://www.researchregistry.com.

## Guarantor

Shingo Ito.

## Provenance and peer review

Not commissioned, externally peer-reviewed.
